# Perceptions and attitudes toward obesity care among nursing professionals

**DOI:** 10.1186/s12912-026-05147-0

**Published:** 2026-08-04

**Authors:** Jana Makuc, Ana Ogrič Lapajne, Špela Hvalec, Mojca Jensterle, Andrej Janež

**Affiliations:** 1 Topolšica Hospital, Topolšica 64, Topolšica, 3326 Slovenia; 2 Community Health Centre Ravne na Koroškem, Ob Suhi 11, Ravne na Koroškem, 2390 Slovenia; 3 Faculty of Medicine, University of Ljubljana, Vrazov trg 2, Ljubljana, 1000 Slovenia; 4 Community Health Centre Idrija, Otona Župančiča 3, Idrija, 5280 Slovenia; 5 Psychiatric Hospital Idrija, Pot sv. Antona 49, Idrija, 5280 Slovenia; 6 University Medical Centre Ljubljana, Zaloška 7, Ljubljana, 1000 Slovenia

**Keywords:** Obesity, Nursing, Bias

## Abstract

**Background:**

The understanding of obesity is evolving from a perceived lifestyle-related condition to a chronic, biologically driven disease. Perception of obesity as a disease among health care professionals is important for effective evidence-based obesity care. Nurses have the most sustained patient contact and play an important role in obesity prevention and management, yet their role in this context remains underexplored. We therefore assessed the perceptions and attitudes towards obesity and obesity care in a Slovenian nursing care cohort.

**Methods:**

Non-interventional, descriptive, cross-sectional study collecting data via an online survey system was distributed to nationwide membership of the Nurses and Midwives Association of Slovenia via their digital communication channels. The online questionnaire was active for three months. Data was integrated with previously obtained responses from physicians, to enable cross-professional comparison.

**Results:**

The majority of respondents agreed that obesity is a disease (82.7%), that is equally important as other chronic diseases (86.9%). Most feel uncomfortable talking about obesity (54.8%) and assume patients fully responsible for their weight (58.1%), which correlates to a lower level in nursing care education. Those with higher nursing care education reported more positive professional attitudes toward obesity (*p* < 0.001). A strong misalignment between professional/personal standpoint was confirmed (*p* < 0.001), however, personal attitude was a strong positive predictor of professional attitude (*p* < 0.001). Female respondents showed more positive attitudes than males (*p* = 0.002). Participants within family medicine model practice (registered nurses with special knowledge in chronic diseases) have the least problems addressing obesity (25%).

**Conclusions:**

The concept of obesity being a disease is not fully adopted in nursing care respondents and a strong misalignment between the professional/personal standpoint exists. Personal attitude and education level were positive predictors of professional attitude towards obesity. Female respondents showed more positive attitudes. These findings highlight the importance of educational interventions aimed not only at increasing professional knowledge but also at addressing personal obesity-related attitudes among nursing professionals.

**Supplementary Information:**

The online version contains supplementary material available at https://doi.org/10.1186/s12912-026-05147-0.

## Introduction

Obesity is a prevalent, complex, progressive and relapsing chronic disease, characterized by abnormal or excessive body fat that impairs health [1]. In a few decades it escalated into a public health challenge [2]. The understanding of obesity has undergone a substantial conceptual shift, evolving from a perceived lifestyle-related condition to a chronic, biologically driven disease [1]. Despite this new knowledge it remains uniquely affected by bias and stigma, extending beyond the general population into healthcare environments. Healthcare professionals/providers (HCPs) of different profiles demonstrate implicit and/or explicit weight bias attitudes toward people with obesity [3]. This is influencing clinical interactions, reducing quality of care, and ultimately affecting health outcomes - contributing to increased morbidity and mortality in people living with obesity independently of weight or body mass index [1, 4]. Studies suggest HCPs should strategically focus on reducing obesity bias and provide high-quality obesity management [1].

In our previous study [5] we assessed the attitude of Slovenian physicians towards obesity and its treatment - suggesting area of expertise related to conscious bias, whereas male gender, younger age, and lower BMI related to unconscious bias toward obesity and its effective care. However, nursing professionals (who often have the most sustained patient contact) remain underexplored in this context. The present study therefore focuses on nursing care providers, assessing how professional knowledge, personal beliefs and demographic characteristics interact in shaping their attitudes toward obesity. Comparison with physician data further positions these findings within a broader healthcare context.

## Materials and methods

### Study design, participants and outcomes

A cross-sectional, non-interventional, descriptive study was employed collecting data via an online survey system (1 ka, GO TEL, Slovenia). Ethical clearance was obtained from the General Hospital Celje Ethics Committee (No. 58/2023/2, issued 5th May 2023) and nationwide distribution was facilitated by the national regulatory authority for nursing and midwifery (the Nurses and Midwives Association of Slovenia) via their digital communication channels. Formal informed consent was waived due to participation being entirely voluntary, optional and anonymous, with no incentives provided. The terms of consent were explained in detail in the keynote and continuation to the questionnaire assumed consent (approved by the Chief Executive Officer of the Nurses and Midwives Association of Slovenia on 27th June 2024).

The survey instrument was derived from a previously used physician questionnaire [5], but underwent targeted modification to reflect nursing-specific roles. Adjustments were concentrated in demographic descriptors (professional titles, education) and clinical management perspectives (trimmed to nursing care). It comprised 27 questions, which included single or multiple choice, numeric response, and ranking formats (an English language version was added as supplementary material).

The link to the online questionnaire was distributed via the Nurses and Midwives Association of Slovenia`s multiple digital channels: twice through their e-newsletter (reaching 14.219 and 14.178 contacts, respectively), once through their Facebook account (reaching 634 contacts) and Instagram account (reaching 173 contacts). The online questionnaire was active for three months (27th June – 24th September 2024). Target sample size was 10% of their full registry. Outcomes were measured by single and multiple item selection, numeric response and ranking.

To enable cross-professional comparison, dataset was integrated with our previously collected physician data [5], forming a unified analytical framework. The questionnaire used in the nursing cohort was directly derived from the physician questionnaire used in our previous study [5] with the wording of all items identical in both professional groups, including both standpoint composites. Since only completely consistent questions were directly compared, the measuring instrument remained the same. Adapted were the demographic descriptors (professional title, workplace) and profession-specific contextual questions, which could not be compared.

### Composite constructs reflecting professional and personal attitudes toward obesity

As with physicians [5], two composite constructs were developed to reflect professional and personal attitudes toward obesity, using the same combination of questions. The constructs were a priori based on theoretical considerations and expert consensus with scoring presented in Tables 1 and 2. In both cases, a higher score indicates evidence-based professional beliefs and less stigmatizing attitudes toward obesity.

**Table 1:** Scoring of professional standpoints on obesity

statement	scoring		
Patients are fully responsible for their weight.	yes (I agree) = 0		no (I disagree) = 1
To lose weight, patients would have to completely change their lifestyle.	yes (I agree) = 0		no (I disagree) = 1
Patients could lose weight if they would make a serious attempt.	yes (I agree) = 0		no (I disagree) = 1
Within the healthcare system, people with overweight or obesity receive (better/same/worse) treatment	better = 0	same = 0.5	worse = 1
Ʃ	0–4		

**Table 2:** Scoring of personal standpoints on obesity

statement	scoring		
When not at work, I find myself using derogatory obesity phrases.	yes (I agree) = 0	no (I disagree) = 1	
People with overweight or obesity do not care for their weight.	yes (I agree) = 0	no (I disagree) = 1	
People with overweight or obesity do not care for their health	yes (I agree) = 0	no (I disagree) = 1	
I relate obesity to pleasent (e.g. good spirit, hedonism)/no extra/unpleasent (e.g. laziness, lack of willpower) feelings	unpleasent = 0	no extra = 1	pleasent = 2
Ʃ	0–5		

The professional standpoint construct was conceptualised as a content-based formative composite index. The selected items were intended to capture complementary aspects of professional attitudes towards obesity, including attribution of responsibility to the patient, treatment expectations and awareness of weight-related inequities within healthcare. Exploratory factor analysis with principal axis factoring and internal consistency estimates were performed descriptively despite the composite conceptualisation to explore the internal structure of the selected items. Complete data were available for 389 participants. Sampling adequacy was modest (KMO = 0.570), while Bartlett’s test of sphericity was significant, χ² = 66.94, *p* < 0.001. One factor had an eigenvalue greater than 1 (1.49). Factor loadings ranged from 0.27 to 0.68, and the extracted factor accounted for 19.3% of the common item variance. Internal consistency was low (Cronbach’s α = 0.411), indicating the composite reflects several interrelated (but not interchangeable) aspects of professional attitudes. As the construct is formative, these reflective indices (factor analysis and Cronbach’s α) are reported for descriptive purposes only; low internal consistency is expected and does not indicate a deficient scale.

The personal standpoint construct was also designed as a composite (formative) index, combining three components of personal attitudes towards obesity - cognitive (beliefs), affective (feelings) and behavioral (actions) [6]. Complete data were available for 390 participants. The suitability of the data for factor analysis was limited, yet acceptable. KMO = 0.506 represents the lower limit of acceptability and the Bartlett test of sphericity was statistically significant (χ² = 127.60; *p* < 0.001), indicating there are statistically significant correlations between the items. As expected for a formative index, internal consistency was low (raw Cronbach’s α = 0.335), and the average inter-item correlation was 0.145, indicating a relatively weak correlation between individual items (in accordance with intentionally measuring different attitude components). As expected, exploratory factor analysis suggested a multidimensional internal structure of the construct - a descriptive Kaiser-criterion extraction (eigenvalue > 1, which tends to over-extract with only four items) revealed two factors (1.529 and 1.154), which together explain approximately 67% of the variance. In the one-factor solution, only the following items significantly saturated: the belief that obese people do not care about their health (factor weight = 0.872), the belief that they do not care about their body weight (factor weight = 0.566), while the items on the use of derogatory terms (0.053) and emotional response (0.120) showed negligible factor weights. This confirms encompassing at least two distinct dimensions (representation of different aspects of personal attitudes toward obesity, rather than a single psychological construct).

### Statistical analysis

Statistical analysis was conducted using SPSS (IBM, version 23.0) and the R programming language (R Core Team, 2021). Descriptive statistics such as mean ± standard deviation (SD), median (Q1-Q3), and frequency (%) were utilized to summarize the data. Normality tests including the Shapiro-Wilk and Kolmogorov-Smirnov tests were performed.

To compare responses between nursing staff groups, genders, and among age and BMI groups, Pearson’s Chi-squared test or Fisher’s Exact test was employed. The Mann-Whitney U test was used to compare overall professional and personal perspectives on obesity between genders. The Kruskal-Wallis rank sum test was applied to compare these perspectives across different HCPs, age groups, and BMI groups. For post hoc analysis, the Dunn test with Bonferroni correction was applied to adjust for multiple comparisons.

Statistical significance was set at *p* < 0.05, using two-tailed tests. Only data from participants who completed the survey were included in the analyses.

Finally, in the combined dataset, a linear regression model with robust standard errors was used to examine the association between professional attitude toward obesity and predictors including type of education, gender, and personal attitude.

## Results

A total of 960 individuals listed in the register of nursing and midwifery care registry opened the survey link, representing a 6.7% response rate to the Nurses and Midwives Association of Slovenia`s invitation. Of these, 460 (47.9%) engaged with the survey, with 396 (41.3%) completing the questionnaire, and 64 providing partial responses. The average completion time was 4 min and 2 s.

### Demographic data

General demographic characteristics (gender, age, BMI, education level) of the nursing care cohort differed significantly in comparison to physicians [5]; *p* < 0.001 for all. Respondents were predominately women (92.8%), age 30–40 (35.0%), with BMI < 25 kg/m2 (41.0%), graduate nurses/midwives (67.1%). A large proportion (33.4%) of respondents refused to provide data on their workplace. Participant characteristics are given in Table 3.

**Table 3:** Participant characteristics

	variable	No.	total
gender	female	362 (92.8%)	392 (100.0%)
	male	28 (7.2%)	
	NA	2	
age (years)	< 30	49 (12.5%)	392 (100.0%)
	30–40	137 (35.0%)	
	41–50	101 (25.8%)	
	51–60	85 (21.7%)	
	> 60	19 (4.9%)	
	NA	1	
professional title	nursing assistant/caregiver or healthcare assistant	128 (32.9%)	392 (100.0%)
	graduate nurse/midwife	261 (67.1%)	
	NA	3	
workplace	the family medicine model practice	20 (7.7%)	392 (100.0%)
	social care institutions	10 (3.8%)	
	primary level of healthcare	92 (35.2%)	
	hospital/medical centre	111 (42.5%)	
	other	28 (10.7%)	
	NA	131	
BMI (kg/m²)	< 25	154 (41.0%)	392 (100.0%)
	25–30	134 (35.6%)	
	31–35	60 (16.0%)	
	36–40	18 (4.8%)	
	> 40	10 (2.7%)	
	NA	16	

Legend: BMI - body mass index, NA - not available (no answer)

### Professional standpoint on obesity and obesity management

Regardless of demographic parameters, majority of respondents agree obesity is a disease (82.7%), equally important as other diseases (86.9%) and know definition of obesity (77.6%). They also believe they could help their patients with losing weight (85.6%) - this was, however, less convincing in participants with lower education (78.4% of nursing assistants/caregivers and HCAs, *p* = 0.004) and those with BMI > 40 kg/m2 (40%, *p* < 0.001). Over three-quarters (78.3%) of respondents are professionally interested in the subject, proportion continuously rising from 71.1% in BMI < 25 kg/m2 to 90.0% in BMI > 40 kg/m2 (*p* = 0.030). Respondents` BMI was the single parameter impacting belief on equal treatment within the healthcare system and the seriousness of patients` approach to treatment: while most respondents believe people living with obesity are treated equally (61.5%) or worse (34.9%), the proportions varied significantly regarding to their BMI (*p* = 0.011). In BMI < 25 kg/m2 the proportions were 66.9% and 26.0%; in BMI > 40 kg/m2 26.0 and 60%, respectively. Also, most respondents (86.7%) believe patients could lose weight if a serious attempt would have been made: those with BMI < 25 kg/m2 agreed in 93.5% and those with BMI > 40 kg/m2 in 55.6% (*p* = 0.003).

More than half of the study participants feel uncomfortable talking about obesity (54.8%) and assume patients fully responsible for their weight (58.1%). A significant correlation to a lower education level was noted in these two questions (nursing assistants/caregivers and HCAs: 70.3%, *p* < 0.001, and 68.8%, *p* = 0.002, respectively). Noteworthy, participants within family medicine model practice have the least problems addressing obesity (25%, *p* = 0.028).

Finally, the combined professional standpoint assessment further confirmed the importance of education level (*p* = 0.028): nursing assistants/caregivers and HCAs scored lower (median 1.0 [0.5;1.5]) in comparison to registered graduate nurs-es/midwives (median 1.5 [0.5;2.0]). The effect of BMI was distinguishable, yet not significant (*p* = 0.053). No other demographic parameter showed significance.

In direct comparison to physicians [5], most answers of the nursing care cohort differed significantly, including obesity being a disease (*p* < 0.001). However, no differences were observed regarding obesity being equally important as other diseases (*p* = 0.455), professional interest in the subject (*p* = 0.290), possibility to help (*p* = 0.070) and belief on equal treatment within the healthcare system (*p* = 0.142).

### Intimate/ personal view on obesity and obesity management

The respondent`s intimate/personal view on obesity being a disease significantly differs from their professional standpoint: 57.7% feel obesity is a disease and 42.3% are convinced to the contrary (*p* < 0.001). Although 48.1% do not engage specific feelings towards obesity, 47.0% associate it with unpleasant feelings (e.g. laziness, lack of willpower) and only 4.9% to pleasant ones (e.g. good spirit, hedonism). Almost one-fifth (19.2%) of participants admitted to using derogatory obesity phrases in their free time. There were no differences in demographic parameters in these questions, but significant differences in direct comparison to physicians [5] (*p* < 0.001, *p* < 0.001 and *p* = 0.032, respectively).

Nevertheless, 81.6% respondents feel that people with normal weight also deal with their body weight, graduate participants more often (*p* = 0.025) in comparison to those with lower nursing care education (85.1% and 75.8%, respectively). Majority disagreed when asked if people with overweight or obesity do not care for their weight and health (no for weight: 92.8%; no for health: 90.6%). There was a gender preference with males (71.4%) being more judgemental (*p* = 0.002). No differences in comparison to physicians [5] were found.

Finally, using composite answers, we found obesity-related personal attitudes were significantly influenced by gender (*p* = 0.002), but not by other demographic parameters. Men scored lower than women: median 2.0 [2.0;3.0] vs. 3.0 [2.0;3.0].

### Practical management

Answers of the nursing care cohort regarding practical management differed significantly (*p* < 0.05) in all aspects in direct comparison to physicians [5]. When considering obesity treatment, nursing care personnel are mostly guided by obesity-related complications (54.0%) and clinical assessment of nutrition (32.5%), and to a lesser ex-tent by BMI (8.2%) or waist circumference (5.4%). Interestingly, participants with lower education were more focused on obesity-related complications (66%) in com-parison to registered graduate participants (47.3%), who were mostly driven by clinical assessment of nutrition (38.8%; *p* = 0.001). There were no other differences regarding demographic parameters.

When asked about obesity complications that are best prevented by losing weight, respondents most commonly recognized cardio-vascular events (93.3%), diabetes (91.5%) and arterial hypertension (91.0%), less often obstructive sleep apnoea syndrome (OSAS; 65.9%), hyperlipidaemia (65.9%) and depression (66.7%). Osteoarthritis was the least recognized (32.6%). Graduate respondents more often recognized OSAS and hyperlipidaemia (*p* = 0.027 and *p* = 0.001, respectively). Respondent`s age was related to recognizing cardio-vascular events (*p* = 0.030), osteoarthritis (*p* = 0.046) and OSAS (*p* = 0.041). Other demographic parameters showed no correlations.

5–15% weight loss was considered as successful for preventing obesity complications/comorbidities in 70.5% of respondents and > 15% weight loss in 25.8% of respondents, regardless of demographic parameters.

When asked about the most effective weight loss method, participants most frequently chose healthy eating (96.9%) and regular physical activity (95.6%) whereas cognitive behavioural therapy (CBT), weight loss medications and metabolic surgery were less frequently chosen (50.3%, 12.8% and 9.7%, respectively). CBT was the only method influenced by demographic parameters: more often chosen by graduate respondents (*p* = 0.036) and rising with age (*p* = 0.002). Most (52.2%) respondents do not feel elderly should be treated differently and have not yet advised obesity management in the local Health Promotion Centre (67.2%). This was done more often in younger (*p* = 0.004), graduate respondents (*p* < 0.001) and nursing care personnel working in secondary healthcare (*p* = 0.001).

Finally, in a merged (doctor` and nursing care) database, a linear regression model with robust standard errors was used to examine the association between professional attitude toward obesity and predictors including type of education, gender, and personal attitude. The model explained approximately 14.7% of the variance in professional attitude scores. Compared to nursing care respondents with basic education, those with higher education in nursing care (β = 0.439, *p* < 0.001) and physicians (β = 0.818, *p* < 0.001) reported significantly more positive professional attitudes toward obesity. Gender was also a significant predictor, with female respondents showing more positive attitudes than males (β = 0.222, *p* = 0.002). Personal attitude toward obesity was a positive predictor of professional attitude (β = 0.260, *p* < 0.001). Detailed responses are given as supplementary material (Table S5).

## Discussion

The concept of obesity being a disease (as opposed to a risk factor) is still not fully adopted [7]. In our nursing care cohort respondents, majority agree obesity is a disease (82.7%), equally important as other diseases (86.9%) and know definition of obesity (77.6%). However, this concept is less accepted in comparison to physicians [5] – regardless of whether they act as representatives of professional healthcare (82.7%) or they reflect their inner beliefs (57.7%; *p* < 0.001 for both) and independent of any demographic parameters. It is also less accepted in comparison to 88% HCPs in ACTION-IO [8]. A strong misalignment between professional/personal standpoint was confirmed (*p* < 0.001). Possibly, this is reflecting the inner dissonance: (in)ability to internalize a belief that is in contrast to the commonly acknowledged simplistic obesity concept [9]. Also, personal attitudes towards obesity are shaped on multiple levels - an individual may simultaneously reject negative stereotypes but still experience certain unpleasant feelings or use socially accepted terms that reflect stigma. Conversely, an individual may express positive feelings towards people with obesity and at the same time believe in stereotypes regarding their personal responsibility for their body weight. This could explain why over three-quarters (78.3%) of respondents are professionally interested in obesity but more than half (54.8%) feel uncomfortable talking about it and assume patients fully responsible for their weight (58.1%). Furthermore, most (86.7%) respondents believed patients could lose weight if a serious attempt would have been made. Attribution of controllability is one of the most well-established predictors of stigma and prejudice - if weight is regarded as changeable through appropriate behavioural choices, people are deemed responsible for their weight [9, 10]. This was also reflected in the psychometric evaluation of the “personal standpoint” composite, where cognitive stereotypes and affective/behavioural responses appeared to represent related but distinct dimensions.

An important observation regarding respondents` BMI and their stance was noted, possibly guided from one’s own feelings and experiences. Respondents` BMI was related to their professional interest in the subject (rising with their BMI; *p* = 0.030), belief on equality within the healthcare system (feeling of equality lowering with increasing BMI; *p* = 0.011), perceiving seriousness of patients` attempt to treatment (believing serious patients attempts increasing with BMI, *p* = 0.003). Respondents with BMI > 40 kg/m2 also showed substantial doubt in their possibility to help their patients with losing weight (40% in comparison to overall 85.6%; *p* < 0.001). We found no data on nursing care providers` BMI impact on obesity care and beliefs; however, in physicians, study showed more normal weight physicians provided recommended obesity care to their patients and felt confident doing so [11]. In this context, weight stigma towards healthcare professionals (possibly influencing practitioner–patient relationship) is vastly under researched; but so far showed no effect based solely on HCPs weight status [12].

Since personal attitude toward obesity was a positive predictor of professional attitude (β = 0.260, *p* < 0.001), changing the obesity narrative should be focused on inner beliefs, possibly through general public perception of obesity. Studies show healthcare professionals exhibit the same levels of implicit bias as the wider population [4]. Currently, high level of confusion is caused by media coverage of overweight/obesity with seemingly contradictory elements, like body positivity and fat shaming, health issues, unrealistic representation [13]. Meta-analysis on weight bias in health care professionals demonstrated implicit and/or explicit weight bias attitudes toward people with obesity held by various profiles of HCPs [3]. Physicians, generally known for collegiality of the guild, exhibited strong antifat physician to physician weight bias - implicit, explicit and professional [14]. Even professionals whose careers emphasize research or the clinical management of obesity show very strong weight bias, indicating pervasive and powerful stigma [15]. In present research, affirmingly, of the participants exhibiting feelings towards obesity, 47.0% associate it with unpleasant and only 4.9% to pleasant ones. Almost one-fifth (19.2%) of participants admitted to using derogatory obesity phrases in their free time, all irrespective of any demographic parameters. Interestingly, significant differences in direct comparison to physicians were noted in these questions – with physicians showing more negative attitudes. Also, males were shown to be more judgmental (*p* = 0.002) - in agreement with previous studies consistently showing lower weight bias in women [5, 16, 17]. This could be related to women being the primary focus of negative media coverage [13].

Despite the obesity-related personal attitudes (unconscious bias), education (level) seems to play an important role in professional attitudes: compared to respondents with basic education, those with higher education in nursing care and physicians reported significantly more positive professional attitudes toward obesity (*p* < 0.001). They were more comfortable talking about obesity (*p* < 0.001) and in a lesser manner assumed patients fully responsible for their weight (*p* = 0.002). Noteworthy, participants within family medicine model practice, equipped with special knowledge in the field of chronic diseases have the least problems addressing obesity (25%, *p* = 0.028). This suggests the importance of focused education with background understanding.

All aspects of the practical management differed significantly (*p* < 0.050) in direct comparison to physicians [5], including recognizing common obesity complications, choosing the most effective weight loss method, obesity management of the elderly. Possibly, this is due to nursing care personnel’s roles being primarily focused on lifestyle interventions (e.g. nutrition and physical activity), anthropometric measurements, health planning, goal setting, supportive care, monitoring progress, and arranging follow-up [18]. Their roles in the management of overweight and obesity are underdeveloped [19]. However, it should be accompanied by sympathetic encouragement to help the client continue despite disappointments and setbacks [20] – stressing the importance of HCPs recognizing their own attitudes/beliefs and consider how they may influence their care delivery [1]. A negative correlation exists between level of implicit bias and indicators of quality of care [4]. Weight bias awareness is, however, low [21]. Medical/nursing schools may reduce students’ weight bias by increasing positive contact between students and patients with obesity and eliminating unprofessional role modelling by faculty members and residents [22]. The new concept offers an opportunity to transform nursing care practice by fostering a holistic, equitable, and evidence-based approach to care [23].

Our study has some limitations. Demographic data of the respondent nursing care cohort differs from our national registry´s data [24], which shows most nursing practitioners are undergraduate (nursing assistants/caregivers and HCAs), up to 29 years old - suggesting this is a non-representative nursing care cohort and therefore the results should not be generalized. Since participants with basic/lower education in nursing care were under-represented, non-response bias must be considered at this point. The response rate to the Nurses and Midwives Association of Slovenia`s invitation was low and understanding the non-response could also be an opportunity for improvement. Lack of motivation to participate could be related to the fact surveys and questionnaires are (too?) often used in nursing research, the questionnaire probably being the most common form of data collection tool used [25, 26]. Confirmed professional interest in obesity in most respondents suggests those who chose to complete the survey may have had greater interest in the topic and/or a more favourable attitudes than non-responders (additional response and/or non-response bias). Distributed online, it is potentially excluding individuals with lack of computer skills and access. Unlikely, but possible, repeated survey entries could exist. Data are self-reported (including the BMI), opening a window for social-desirability bias, although questionnaire design was deliberately offering participants the opportunity to delineate their professional/personal standpoints.

Since a substantial proportion of the variability in our regression model remains unexplained, it is likely that additional factors influencing professional attitude toward obesity were not captured. Also, the associations do not imply causality (cross-sectional study design). However, these factors should be further identified – possibly systemic limitations such as lack of proper equipment for diagnosing, treating and taking care of patients with obesity or communication soft skills [27].

## Conclusions

The concept of obesity being a disease is still not fully adopted in nursing care respondents and a strong misalignment between the professional/personal standpoint exists. Personal attitude and education level were positive predictors of professional attitude towards obesity. Female respondents showed more positive attitudes. These findings highlight the importance of educational interventions aimed not only at increasing professional knowledge but also at addressing personal obesity-related attitudes among nursing professionals.

## Supplementary Information

Below is the link to the electronic supplementary material.

Supplementary Material 1

Supplementary Material 2

## Data Availability

Research data are newly generated. They were collected through and deposited in the 1KA application, available at https://www.1ka.si/d/sl. Data access is available on request from the corresponding author due to participants’ privacy.

## Abbreviations

HCP: Healthcare professional/provider
HA: Healthcare Assistant
OSAS: Obstructive sleep apnoea syndrome
CBT: Cognitive behavioural therapy

## References

[1] Wharton S, Lau DCW, Vallis M, et al. Obesity in adults: a clinical practice guideline. CMAJ. 2020;192(31):E875–91. 10.1503/cmaj.191707. 32753461 PMC7828878

[2] World Health Organization. Fact Sheet: Obesity and overweight [Internet]. [cited 2026 Jan 25]. Available from: https://www.who.int/news-room/fact-sheets/detail/obesity-and-overweight.

[3] Lawrence BJ, Kerr D, Pollard CM, et al. Weight bias among health care professionals: A systematic review and meta-analysis. Obes (Silver Spring). 2021;29(11):1802–12. 10.1002/oby.23266. 34490738

[4] FitzGerald C, Hurst S. Implicit bias in healthcare professionals: a systematic review. BMC Med Ethics. 2017;18(1):19. 10.1186/s12910-017-0179-8. Published 2017 Mar 1. 28249596 PMC5333436

[5] Makuc J, Ogrič Lapajne A, Hvalec Š, Jensterle M, Janež A. Factors Affecting the Unconscious Bias of Healthcare Professionals in Obesity Care. J Clin Med. 2025;14(5):1486. 10.3390/jcm14051486. Published 2025 Feb 23. 40094928 PMC11900521

[6] Eagly AH, Chaiken S. The advantages of an inclusive definition of attitude. Soc Cogn. 2007;25(5):582–602. 10.1521/soco.2007.25.5.582.

[7] Luli M, Yeo G, Farrell E et al. The implications of defining obesity as a disease: a report from the association for the study of obesity 2021 annual conference. EClinicalMedicine. 2023;58:101962 10.1016/j.eclinm.2023.101962. Published 6 Apr 2023. PMC10119881 37090435

[8] Caterson ID, Alfadda AA, Auerbach P, et al. Gaps to bridge: Misalignment between perception, reality and actions in obesity. Diabetes Obes Metab. 2019;21(8):1914–24. 10.1111/dom.13752. 31032548 PMC6767048

[9] Busetto L, Sbraccia P, Vettor R. Obesity management: at the forefront against disease stigma and therapeutic inertia. Eat Weight Disord. 2022;27(2):761–8. 10.1007/s40519-021-01217-1. 34052990 PMC8933346

[10] Hoyt CL, Burnette JL, Thomas FN, Orvidas K. Public Health Messages and Weight-Related Beliefs: Implications for Well-Being and Stigma. Front Psychol. 2019;10:2806. 10.3389/fpsyg.2019.02806. Published 2019 Dec 17. 31920849 PMC6928046

[11] Bleich SN, Bennett WL, Gudzune KA, Cooper LA. Impact of physician BMI on obesity care and beliefs. Obes (Silver Spring). 2012;20(5):999–1005. 10.1038/oby.2011.402. PMC3645927 22262162

[12] Čadek M, Täuber S, Lawrence BJ, Flint SW. Effect of health-care professionals’ weight status on patient satisfaction and recalled advice: a prospective cohort study. EClinicalMedicine. 2023;57:101855. 10.1016/j.eclinm.2023.101855. Published 2023 Feb 16. 36864980 PMC9971267

[13] Obesity Health Alliance [Internet]. [cited 2026 Jan 25]. Available from: https://obesityhealthalliance.org.uk/wp-content/uploads/2022/04/Obesity-Insight-Research.pdf

[14] McLean ME, McLean LE, McLean-Holden AC, et al. Interphysician weight bias: A cross-sectional observational survey study to guide implicit bias training in the medical workplace. Acad Emerg Med. 2021;28(9):1024–34. 10.1111/acem.14269. 33914377

[15] Schwartz MB, Chambliss HO, Brownell KD, Blair SN, Billington C. Weight bias among health professionals specializing in obesity. Obes Res. 2003;11(9):1033–9. 10.1038/oby.2003.142. 12972672

[16] Sabin JA, Marini M, Nosek BA. Implicit and explicit anti-fat bias among a large sample of medical doctors by BMI, race/ethnicity and gender. PLoS ONE. 2012;7(11):e48448. 10.1371/journal.pone.0048448. 23144885 PMC3492331

[17] Baska A, Świder K, Zgliczyński WS, Kłoda K, Mastalerz-Migas A, Babicki M. Is Obesity a Cause for Shame? Weight Bias and Stigma among Physicians, Dietitians, and Other Healthcare Professionals in Poland-A Cross-Sectional Study. Nutrients. 2024;16(7):999. 10.3390/nu16070999. Published 2024 Mar 29. 38613032 PMC11013468

[18] Piwowarczyk E, MacPhee M, Howe J. Nurses’ Role in Obesity Management in Adults in Primary Healthcare Settings Worldwide: A Scoping Review. Healthc (Basel). 2024;12(17):1700. 10.3390/healthcare12171700. Published 2024 Aug 26. PMC11395003 39273724

[19] Sen A, Ho KYA, Goh WKF, Chew HSJ. The roles of nurses in preventing and managing excess weight among adults: A systematic scoping review. Nurs Outlook. 2025;73(2):102377. 10.1016/j.outlook.2025.102377. 39933258

[20] Coutts A. The nurse’s role in providing strategies and advice on weight management. Br J Nurs. 2021;30(21):S20–7. 10.12968/bjon.2021.30.21.S20. 34839689

[21] Miller DP Jr, Spangler JG, Vitolins MZ, et al. Are medical students aware of their anti-obesity bias? Acad Med. 2013;88(7):978–82. 10.1097/ACM.0b013e318294f817. 23702519 PMC3930920

[22] Phelan SM, Puhl RM, Burke SE, et al. The mixed impact of medical school on medical students’ implicit and explicit weight bias. Med Educ. 2015;49(10):983–92. 10.1111/medu.12770. 26383070 PMC4755318

[23] Nashwan AJ. Redefining Obesity: Implications for Holistic Nursing Practice. Cureus. 2025;17(1):e78134. 10.7759/cureus.78134. Published 2025 Jan 28. 40018480 PMC11867199

[24] Nurses and Midwives Association of Slovenia. Analiza razmer na trgu dela in predlog politik ter ukrepov kadrovske strategije v dejavnosti zdravstvene in babiške nege [Internet]. [cited 2026 Jan 25]. Available from: https://zbornica-zveza.si/wp-content/uploads/2025/03/Analiza_razmer_na_trgu_dela_in_predlog_politik_ter_ukrepov_kadrovske_strategije2.pdf.

[25] Murray P. Fundamental issues in questionnaire design. Accid Emerg Nurs. 1999;7(3):148–53. 10.1016/s0965-2302(99)80074-5. 10693384

[26] Timmins F. Surveys and questionnaires in nursing research. Nurs Stand. 2015;29(42):42–50. 10.7748/ns.29.42.42.e8904. 26080989

[27] Sobczak K, Leoniuk K. Attitudes of Medical Professionals Towards Discrimination of Patients with Obesity. Risk Manag Healthc Policy. 2021;14:4169–75. 10.2147/RMHP.S317808. 34675711 PMC8504468

