# Supplementary material for: Perceptions and attitudes toward obesity care among nursing professionals
Source: BMC Nurs. 2026 Aug 4;25:854. doi: 10.1186/s12912-026-05147-0 (PMC13584392; doi:10.1186/s12912-026-05147-0)
Supplement: Supplementary file 1 [file 12912_2026_5147_MOESM1_ESM.docx]

**Supplementary Materials:**

**Obesity – Attitudes of Slovenian Nursing Professionals (questionnaire)**

**Table S1.** Section 1 - Basic demographic information

| **No.** | **question** | **answer** |
| --- | --- | --- |
| **Q1** | **Your professional title:** | - graduate nurse/midwife |
|  |  | - nursing assistant/caregiver or healthcare assistant |
| **Q2** | **Your workplace:** | - the family medicine model practice - social care institutions - primary level of healthcare - hospital/medical centre - other |
| **Q3** | **Your gender:** | - male |
|  |  | - female |
| **Q4** | **Your age:** | - <30 years |
|  |  | - 30-40 years |
|  |  | - 41-50 years |
|  |  | - 51-60 years |
|  |  | - > 60 year |
| **Q5** | **Your body mass index (BMI)** (kg/m²): | - < 25 |
|  |  | - 25-30 |
|  |  | - 31-35 |
|  |  | - 36-40 |
|  |  | - >40 |
|  |  | - I prefer not to answer |

**Table S2.** Section 2 - What is your PROFESSIONAL stance?

| **No.** | **statement** | | | | |
| --- | --- | --- | --- | --- | --- |
| **Q6** | **As a healthcare professional, I believe/assess/agree…** | obesity is a disease | | yes | no |
| **Q7** |  | I am familiar with the definition of obesity | | yes | no |
| **Q8** | **Your attitude towards patients living with overweight or obesity:** | A patient’s body weight is entirely their own responsibility^†^ | | yes | no |
| **Q9** |  | To lose weight, patients must completely change their lifestyle^†^ | | yes | no |
| **Q10** |  | Patients could lose weight if they really set their mind to it^†^ | | yes | no |
| **Q11** |  | I feel uncomfortable discussing my patients’ weight unless they mention it first^†^ | | yes | no |
| **Q12** |  | I consider obesity to be less important than other diseases^†^ | | yes | no |
| **Q13** |  | I cannot help patients lose weight | | yes | no |
| **Q14** | **I am interested in the field of obesity professionally** | | | yes | no |
| **Q15** | **I believe that people with overweight or obesity receive:** | | - the same treatment as everyone else | | |
|  |  |  | - worse treatment | | |
|  |  |  | - better treatment | | |

^†^question from or similar to the ACTION-IO study

**Table S3.** Section 3 - What is your PERSONAL attitude toward obesity?

| **No.** | **statement** | | | |
| --- | --- | --- | --- | --- |
| **Q16** | **I associate obesity with:** | - positive feelings (e.g., good mood, hedonism) | | |
|  |  | - negative feelings (e.g., laziness, lack of willpower) | | |
|  |  | - no particular feelings | | |
| **Q17** | **I personally think of obesity as a disease** | | yes | no |
| **Q18** | **Outside of work, I catch myself using derogatory terms related to obesity in everyday conversations** | | yes | no |
| **Q19** | **Normally nourished individuals do not worry about their body weight** | | yes | no |
| **Q20** | **People with overweight or obesity do not care about their body weight** | | yes | no |
| **Q21** | **People with overweight or obesity do not care about their health** | | yes | no |

**Table S4.** Section 4 - Treatment

| **No.** | **statement** | | | |
| --- | --- | --- | --- | --- |
| **Q22** | **When planning obesity treatment, the most important**  **factor for me is:** | - body mass index | | |
|  |  | - waist circumference | | |
|  |  | - clinical assessment of nutritional status | | |
|  |  | - obesity-related complications | | |
| **Q23** | **Which complications do you think are best prevented by weight reduction?**  (multiple choice) | - diabetes | | |
|  |  | - arterial hypertension | | |
|  |  | - hyperlipidemia | | |
|  |  | - cardiovascular events | | |
|  |  | - osteoarthritis | | |
|  |  | - depression | | |
|  |  | - sleep apnea syndrome | | |
| **Q24** | **What weight loss percentage do you consider successful for preventing or treating complications and associated diseases?** | - <5% | | |
|  |  | - 5-15% | | |
|  |  | - >15% | | |
| **Q25** | **The most effective therapeutic approach for treating obesity is:**  (multiple choice) | - regular physical activity | | |
|  |  | - healthy nutrition | | |
|  |  | - behavioral cognitive therapy | | |
|  |  | - pharmacotherapy | | |
|  |  | - metabolic surgery | | |
| **Q26** | **In my clinical practice, I have recommended treatment at a health promotion center for a person with obesity** | | yes | no |
| **Q27** | **Is your approach to managing obesity in elderly patients different?** | | yes | no |
