# Supplementary material for: Perceptions and attitudes toward obesity care among nursing professionals
Source: BMC Nurs. 2026 Aug 4;25:854. doi: 10.1186/s12912-026-05147-0 (PMC13584392; doi:10.1186/s12912-026-05147-0)
Supplement: Supplementary file 2 [file 12912_2026_5147_MOESM2_ESM.docx]

**Supplementary Materials:**

**Table S5**: Comparison (doctor` and nursing care) database

|  | | **variable** | **profession** | | **total** | p value |
| --- | --- | --- | --- | --- | --- | --- |
|  |  |  | **nurse** (N) | **doctor** (N) |  |  |
| **Q6** | As a healthcare professional, I agree obesity is a disease | yes | 320 | 733 | 1181 | <0.0001 |
|  |  | no | 67 | 50 |  |  |
|  |  | NA | 5 | 6 |  |  |
| **Q7** | As a healthcare professional, I am familiar with the definition of obesity | yes | 288 | 652 | 1181 | 0.0128 |
|  |  | no | 83 | 127 |  |  |
|  |  | NA | 21 | 10 |  |  |
| **Q8** | A patient’s body weight is entirely their own responsibility | yes (agree) | 226 | 329 | 1181 | <0.0001 |
|  |  | no (disagree) | 163 | 453 |  |  |
|  |  | NA | 3 | 7 |  |  |
| **Q9** | To lose weight, patients must completely change their lifestyle | yes (agree) | 350 | 664 | 1181 | 0.0247 |
|  |  | no (disagree) | 42 | 122 |  |  |
|  |  | NA | 0 | 3 |  |  |
| **Q10** | Patients could lose weight if they really set their mind to it^†^ | yes (agree) | 338 | 641 | 1181 | p 0.0410 |
|  |  | no (disagree) | 52 | 141 |  |  |
|  |  | NA | 2 | 7 |  |  |
| **Q11** | I feel uncomfortable discussing my patients’ weight unless they mention it first^†^ | yes (agree) | 213 | 224 | 1181 | <0.0001 |
|  |  | no (disagree) | 176 | 555 |  |  |
|  |  | NA | 3 | 10 |  |  |
| **Q12** | I consider obesity to be less important than other diseases | yes (agree) | 51 | 90 | 1181 | 0.4554 |
|  |  | no (disagree) | 339 | 688 |  |  |
|  |  | NA | 2 | 11 |  |  |
| **Q13** | I cannot help patients lose weight | yes (agree) | 56 | 145 | 1181 | 0.0704 |
|  |  | no (disagree) | 332 | 631 |  |  |
|  |  | NA | 4 | 13 |  |  |
| **Q14** | I am interested in the field of obesity professionally | yes | 307 | 593 | 1181 | 0.2902 |
|  |  | no | 85 | 192 |  |  |
|  |  | NA | 0 | 4 |  |  |
| **Q15** | I believe that people with overweight or obesity receive: | same treatment | 14 | 47 | 1181 | 0.1419 |
|  |  | worse treatment | 241 | 452 |  |  |
|  |  | better treatment | 137 | 290 |  |  |
| **Q16** | I associate obesity with: | negative feelings | 183 | 389 | 1181 | <0.0001 |
|  |  | no particular feelings | 190 | 364 |  |  |
|  |  | positive feelings | 19 | 34 |  |  |
|  |  | NA | 0 | 2 |  |  |
| **Q17** | I personally think of obesity as a disease | yes | 224 | 582 | 1181 | <0.0001 |
|  |  | no | 164 | 205 |  |  |
|  |  | NA | 4 | 2 |  |  |
| **Q18** | Outside of work, I catch myself using derogatory terms related to obesity in everyday conversations | yes | 75 | 195 | 1181 | 0.0323 |
|  |  | no | 316 | 593 |  |  |
|  |  | NA | 1 | 1 |  |  |
| **Q19** | Normally nourished individuals do not worry about their body weight | yes (agree) | 72 | 138 | 1181 | 0.7177 |
|  |  | no (disagree) | 320 | 650 |  |  |
|  |  | NA | 0 | 1 |  |  |
| **Q20** | People with overweight or obesity do not care about their body weight | yes (agree) | 28 | 49 | 1181 | 0.5410 |
|  |  | no (disagree) | 363 | 738 |  |  |
|  |  | NA | 1 | 2 |  |  |
| **Q21** | People with overweight or obesity do not care about their health | yes (agree) | 37 | 72 | 1181 | 0.8661 |
|  |  | no (disagree) | 355 | 716 |  |  |
|  |  | NA | 0 | 1 |  |  |
| **Q22** | When planning obesity treatment, the most important  factor for me is: | BMI | 32 | 107 | 1181 | <0.0001 |
|  |  | clinical assessment of nutritional status | 127 | 159 |  |  |
|  |  | waist circumference | 21 | 74 |  |  |
|  |  | obesity-related complications | 211 | 446 |  |  |
|  |  | NA | 1 | 3 |  |  |
| **Q23** | Which complications do you think are best prevented by weight reduction?  (multiple choice) | diabetes | 357 (91%) | 754 (95.6%) | 1181 | 0.0038 |
|  |  | arterial hypertension | 355 (90.6%) | 680 (86.2%) |  | 0.0193 |
|  |  | hyperlipidemia | 257 (65.6%) | 590 (74.8%) |  | 0.0013 |
|  |  | cardiovascular events | 364 (92.9%) | 716 (90.7%) |  | 0.1485 |
|  |  | osteoarthritis | 127 (32.4%) | 406 (51.5%) |  | <0.0001 |
|  |  | depression | 260 (66.3%) | 423 (53.6%) |  | <0.0001 |
|  |  | sleep apnea syndrome | 257 (65.6%) | 632 (80.1%) |  | <0.0001 |
| **Q24** | What weight loss percentage do you consider successful for preventing or treating complications and associated diseases? | >15% | 100 | 259 | 1181 | <0.0001 |
|  |  | <5% | 14 | 22 |  |  |
|  |  | 5-15% | 273 | 486 |  |  |
|  |  | NA | 5 | 22 |  |  |
| **Q25** | The most effective therapeutic approach for treating obesity is:  (multiple choice) | regular physical activity | 373 (95.1%) | 704 (89.2%) | 1077 (91.3%) | 0.0002 |
|  |  | healthy nutrition | 378 (96.2%) | 717 (90.9%) | 1095 (92.9%) | 0.0001 |
|  |  | behavioral cognitive therapy | 196 (49.9%) | 491 (62.2%) | 687 (58.3%) | 0.0001 |
|  |  | pharmacotherapy | 50 (12.7%) | 229 (29.0%) | 279 (23.7%) | <0.0001 |
|  |  | metabolic surgery | 38 (9.7%) | 168 (21.3%) | 206 (17.5%) | <0.0001 |
| **Q26** | In my clinical practice, I have recommended treatment at a health promotion center for a person with obesity | yes | 128 | 403 | 1181 | <0.0001 |
|  |  | no | 262 | 382 |  |  |
|  |  | NA | 2 | 4 |  |  |
| **Q27** | Is your approach to managing obesity in elderly patients different? | yes | 186 | 418 | 1181 | 0.0626 |
|  |  | no | 203 | 362 |  |  |
|  |  | NA | 3 | 9 |  |  |
| **professional standpoint**  **composit** | | Min / Max | 0 / 4.0 | 0 / 4.0 | 0 / 4.0 | <0.0001 |
|  |  | Med [IQR] | 1.0 [0.5;2.0] | 1.5 [0.5;2.0] | 1.5 [0.5;2.0] |  |
|  |  | Mean (std) | 1.3 (0.9) | 1.6 (0.9) | 1.5 (0.9) |  |
|  |  | N (NA) | 388 (4) | 778 (11) | 1166 (15) |  |
| **professional standpoint**  **composit** | | Min / Max | 0 / 5.0 | 0 / 5.0 | 0 / 5.0 | 0.2371 |
|  |  | Med [IQR] | 3.0 [3.0;4.0] | 3.0 [3.0;4.0] | 3.0 [3.0;4.0] |  |
|  |  | Mean (std) | 3.2 (0.9) | 3.1 (1.0) | 3.2 (0.9) |  |
|  |  | N (NA) | 390 (2) | 783 (6) | 1173 (8) |  |
